# Development of a targeted client communication intervention to women using an electronic maternal and child health registry: a qualitative study

**DOI:** 10.1186/s12911-019-1002-x

**Published:** 2020-01-06

**Authors:** Binyam Bogale, Kjersti Mørkrid, Brian O’Donnell, Buthaina Ghanem, Itimad Abu Ward, Khadija Abu Khader, Mervett Isbeih, Michael Frost, Mohammad Baniode, Taghreed Hijaz, Tamara Awwad, Yousef Rabah, J. Frederik Frøen

**Affiliations:** 1 Global Health Cluster, Division for Health Services, Norwegian Institute of Public Health, Oslo, Norway; 2 Center for Intervention Science in Maternal and Child Health (CISMAC), University of Bergen, Bergen, Norway; 3 The Palestinian National Institute of Public Health, World Health Organization, Ramallah, Palestine; 4 Health Information Systems Program, Department of Informatics, University of Oslo, Oslo, Norway; 5 The Palestinian Ministry of Health, Ramallah, Palestine

**Keywords:** Targeted client communication, Digital health, mHealth, SMS, Text messages, Antenatal care, Maternal and child health, Electronic registry, DHIS2

## Abstract

**Background:**

Targeted client communication (TCC) using text messages can inform, motivate and remind pregnant and postpartum women of timely utilization of care. The mixed results of the effectiveness of TCC interventions points to the importance of theory based interventions that are co-design with users. The aim of this paper is to describe the planning, development, and evaluation of a theory led TCC intervention, tailored to pregnant and postpartum women and automated from the Palestinian electronic maternal and child health registry.

**Methods:**

We used the Health Belief Model to develop interview guides to explore women’s perceptions of antenatal care (ANC), with a focus on high-risk pregnancy conditions (anemia, hypertensive disorders in pregnancy, gestational diabetes mellitus, and fetal growth restriction), and untimely ANC attendance, issues predefined by a national expert panel as being of high interest. We performed 18 in-depth interviews with women, and eight with healthcare providers in public primary healthcare clinics in the West Bank and Gaza. Grounding on the results of the in-depth interviews, we used concepts from the Model of Actionable Feedback, social nudging and Enhanced Active Choice to compose the TCC content to be sent as text messages. We assessed the acceptability and understandability of the draft text messages through unstructured interviews with local health promotion experts, healthcare providers, and pregnant women.

**Results:**

We found low awareness of the importance of timely attendance to ANC, and the benefits of ANC for pregnancy outcomes. We identified knowledge gaps and beliefs in the domains of low awareness of susceptibility to, and severity of, anemia, hypertension, and diabetes complications in pregnancy. To increase the utilization of ANC and bridge the identified gaps, we iteratively composed actionable text messages with users, using recommended message framing models. We developed algorithms to trigger tailored text messages with higher intensity for women with a higher risk profile documented in the electronic health registry.

**Conclusions:**

We developed an optimized TCC intervention underpinned by behavior change theory and concepts, and co-designed with users following an iterative process. The electronic maternal and child health registry can serve as a unique platform for TCC interventions using text messages.

## Background

The introduction of information and communication technologies in the health sector is revolutionizing health information communication [1, 2]. Digital health applications, such as Short Message Services (SMS), are increasingly common, including in resource-limited settings [3–5]. Targeted Client Communication (TCC) via SMS is being used to improve people’s knowledge and attitudes towards healthy behaviors and utilization of healthcare services [6–9]. TCC, in this context, is defined as transmission of unidirectional targeted health content (pregnancy related information and appointment reminders) tailored to individuals, based on static and dynamic information about recipients from a routine maternal and child health registry system (adapted from World Health Organization [3], and Agarwal et al. [10]).

Well-informed clients can participate in the decision making processes of their own clinical care, and are empowered to make informed health decisions in dialog with their healthcare providers [11]. Therefore, well-informed clients, have the potential to improve the quality content of care they receive. Simple appointment reminders, alone or with health promotion content via SMS, have the potential to boost antenatal care (ANC), postnatal care (PNC), and newborn care service utilization [8, 12–16], but the effectiveness is mixed [17, 18].

Development of an effective behavior change intervention, including TCC intervention, is complex and requires careful design, implementation and evaluation [19]. Despite the growing number of TCC interventions via SMS [20], recent systematic review found that most of them are *“under-theorized, poorly specified and vaguely described*” [14], and the process of end-user involvement is seldom reported [17]. TCC interventions are more effective if the assessment of the current behavior, the identification of gaps between the current and preferred behavior, and the approach to bridge these gaps are grounded on sound behavioral change theories [13, 14, 17, 18]. Theory based interventions are often specific and replicable due to the theory driven description of the design, development and implementation processes [21–23]. Behavior change theories, such as the Health Belief Model (HBM) [24] can be used to assess the context and guide development and evaluation of TCC interventions [22].

The text message content in TCC interventions via SMS, tends to be generic and is rarely tailored towards important individual characteristics [7, 8, 14]. Most health campaigns, especially in low-and middle-income countries, recruit recipient on-demand and gather a few background characteristics [3]. This approach may have limited the availability of information about the recipient, and thus make the development of individualized messages difficult. Furthermore, most digital maternal and child health interventions are without a focus on continuity of care [7]. Developing and sending TCC interventions from a longitudinal registry environment, that includes dynamic information over the course of pregnancy and delivery, to our knowledge, has not been reported previously. The national electronic maternal and child health registry (MCH eRegistry) in Palestine, built on the District Health Information System 2 (DHIS2) software [25], provides such an opportunity [26, 27]. Healthcare providers enter individual-level patient information into the eRegistry at the point of care. The systematic, uniform and longitudinally collected data generate algorithms that drive the eRegistry, including TCC via SMS, which can increase timely attendance and the quality of ANC and PNC [26].

Following Abroms’ [28] recommendation of setting goals for TCC interventions, our goal was to encourage pregnant and postpartum women to attend scheduled visits in a timely manner, and empower them to come with expectations, demand services, and make informed health choices. Therefore, TCC intervention can improve the effective coverage of ANC and PNC services by influencing both the utilization of services (coverage) and quality (appropriate quality contents and timeliness of essential interventions) [29, 30].

This paper presents a theory-driven process from conceptualization and contextual assessment, to development and evaluation of a tailored TCC intervention using individual background characteristics from the MCH eRegistry in Palestine. We used recommended steps for developing and evaluating text messages [28] and relevant components of related reporting checklist, mERA [31] and TIDieR [32] throughout. The intervention described in this paper has subsequently been implemented in Palestine as a cluster-randomized controlled effectiveness trial (ISRCTN10520687).

## Methods

### Setting

We conducted this research to prepare a TCC intervention for a cluster-randomized trial (trial registration No: ISRCTN10520687), embedded in the national MCH eRegistry in the public primary healthcare clinics in Palestine [26, 27]. Approximately 50% of women in Palestine utilize public primary healthcare clinics for ANC and PNC services [33]. The organizational structure of the public primary healthcare clinics is reported elsewhere [27, 34]. The female literacy rate is above 94% [35], and approximately 85% of the women registered in the MCH eRegistry, have provided a mobile phone number.

A national expert panel identified anemia, hypertensive disorders of pregnancy (HDP), gestational diabetes mellitus (GDM), fetal growth restriction (FGR), and untimely ANC attendance as priority issues, on which we concentrated our efforts.

### Theoretical framework and data collection instruments

We used the HBM [24] to guide our interviews and assessments of women’s beliefs and perceptions towards the prioritized high-risk conditions described above. We developed two in-depth interview guides, one for pregnant women and one for healthcare providers, to explore all six constructs (perceived susceptibility, perceived severity, perceived benefits, perceived barriers, cue to action, self-efficacy) of the HBM [24]. The HBM constructs were consolidated into three domains: 1) women’s perceptions of personal risks for the high-risk conditions (susceptibility and severity), 2) perception of benefits of attending ANC for those high-risk conditions, and 3) factors influencing the decision to attend timely ANC (perceived barriers, cues-to-action, and self-efficacy). The interview guides also included additional questions regarding health information sources, counseling, and views on the use of SMS to address knowledge gaps. The English interview guides were translated to Arabic and back-translated to English by an external person.

### Sampling and data collection process

We purposively selected seven public primary healthcare clinics where both pregnant women and healthcare providers were invited to participate in the study. Women, who attended ANC at the day of data collection, were asked to take part in the study by means of convenience sampling. We obtained oral informed consent from all informants. In total, four trained female nurses conducted 15 in-depth interviews in a private room, and three phone-interviews with pregnant women who never returned for ANC to the public primary healthcare clinic where they were first registered. The interviewers also conducted in-depth interviews with four MCH doctors and four nurses/midwives. We stopped further interviews after reaching theoretical saturation. All interviews were audio-recorded.

### Data analysis

We transcribed the interviews and translated them to English. NVivo 12 software (QSR International Pty Ltd. Version 12, 2018) was used for all data management and subsequent analyses. We performed thematic analyses according to HBM constructs. First, we categorized the texts into nodes, referring to the HBM constructs, and then performed line-by-line coding. The main themes were developed and named from codes under each node. We also thematically analyzed information unrelated to the HBM. Three researchers participated in defining the themes based on the data.

### Composing and evaluating the text messages

We used the results of the in-depth interviews to compose the TCC content to be sent via SMS. The TCC content was developed to fill the major awareness and knowledge gaps related to the three HBM constructs (Table 2). The prioritized conditions were linked with the recommended timing of screenings at each of the five sentinel ANC visits, according to the national guidelines (Additional file 1). We used the Model of Actionable Feedback [36] and concepts of social nudging and Enhanced Active Choice [11] to compose the TCC content (Additional file 2). We did not state the recipient’s risk factors to ensure confidentiality. The TCC content was composed in English, translated to Arabic, and independently back-translated to English. We gathered feedback on the TCC content in a stepwise manner, and incorporated comments prior to the next round of feedback. The first consultation with national health education experts on the TCC content suggested further contextualization such as using local expressions. We interviewed healthcare providers and pregnant women to assess the TCC content’s understandability and acceptance, in addition to the preferred time of the day to receive the text messages. The final text message library was created after considering all comments.

### Technology platform

We wrote algorithms to trigger each text message types in the text message library after determining the parameters that characterize the eligibility of who should receive which specific text message. All these parameters are available as a data point in the MCH eRegistry, to mention a few that are included, gestational age, date of scheduled visit, various risk factors, risk conditions, and assent to receive the text message. The algorithms run at each interaction with the data points and sends or schedules messages accordingly after inserting the name, scheduled date, and the name of clinic in to the predesigned message template. The scheduler is set to run automatically at agreed up on time with the users on daily basis to send the scheduled messages after linking to the recipients’ phone number. We use the DHIS2 Tracker App [25] to send messages from the MCH eRegistry through a local telecom service provider’s SMS gateway, as per agreement with the Ministry of Health.

## Results

We first present the findings from the in-depth interviews with pregnant women and healthcare providers and second, how we utilized these findings in iterative rounds of development and evaluation of the TCC intervention with users.

### Part I: findings from in-depth interviews

Among 18 interviewed pregnant women, seven had a high-risk condition in the current pregnancy, and seven were primigravida. The participants mean age was 26 years, ranging from 20 to 33 years. All interviewed women had formal education and seven of them held a college degree or above.

We present pregnant women’s perceptions of the prioritized high-risk conditions (anemia, HDP, GDM, and FGR) and untimely ANC attendance using the HBM (Table 1). We also present results from healthcare providers’ perspectives and experiences, where relevant.

**Table 2:** Content creation for the identified constructs of the Health Belief Model and the application of selected concepts to structure and frame messages, an example

Targeted HBM constructs	Gaps and considerations	Source of information	Example phrases
Perceived Susceptibility	• Specifying risks to pregnancy	• Finding from part I	1 in 20 develop high blood pressure in pregnancy.
	• Statistics	• Nudging concept	
	• Scaled intensity: more messages to those with risk-factors	• MAF • Nudging concept	
Perceived Severity	• Consequences to the baby and the women herself	• Findings from part I	This can affect the baby’s nutrition and growth. If not measured and managed, it can affect your health too.
	• No mentioning of severe/grave consequences	• EAC and MAF	
Perceived Benefits	• Guideline based available screening services at the PHCs	• Mapping: ANC guideline	We will measure your blood pressure and proteins in your urine that can be a sign of high blood pressure.
	• Specifying beneficial test beforehand Personalization	• Findings from part I • EAC	
	• Timed to the benefit	• MAF and EAC	

*HBM* Health Belief Model, *MAF* Model of Actionable Feedback, *EAC* Enhanced Active Choice

### Perceptions of high-risk conditions and timely attendance

#### Perception of susceptibility and severity

Pregnant women, in general, perceived that they had low susceptibility to the high-risk conditions, and that these had low severity. Women with knowledge of the high risk conditions, a history of pregnancy complications, or knew someone with a history of pregnancy complications, perceived greater susceptibility to the high-risk conditions compared with their peers. Women engaged in self-care activities, such as healthy diet, exercise, and regular checkups, perceived themselves as less susceptible to pregnancy complications compared with women not engaged in self-care activities. Most pregnant women knew the general consequences and complications of anemia, diabetes, and hypertension as chronic diseases for the general population, but not their effects in pregnancy on maternal and fetal outcomes (Table 1).

#### Perceived benefits of timely ANC attendance

Pregnant women recognized the benefits of attending ANC on wellbeing, both for their baby and themselves. However, they had little awareness of the importance of timely ANC attendance for appropriate screening and management. The pregnant women’s perceived benefits of attending ANC for screening and management of high-risk conditions were affected by individual background characteristics. Women identified with a high-risk condition or with a history of a pregnancy complication, attended ANC more often than women without any current or previous complications. These women were also more aware of what to expect during ANC visits and the importance of timely attendance. Primigravida women were eager to attend ANC, but had low awareness of what to expect regarding screening and management activities, and the importance of timely attendance. Healthcare providers reported that they provided attractive ANC services to women that contributed positively to women’s ANC attendance (Table 1).

#### Perceptions about barriers, cues-to-action, and self-efficacy

In general, women’s perceptions of barriers, lack of cues-to-action, or lack of self-efficacy were not main factors preventing women from attending ANC services. Among women interviewed, the majority reported that accessibility and lack of support from the husband and/or other family members were not a problem. However, a few women with young children stated that lack of support in childcare was a barrier for them to attend ANC. Low perceived benefits from ANC attendance, along with low perceptions of susceptibility to, and severity of, the high-risk conditions, were the main barriers to timely ANC attendance.

Most women attended ANC despite the lack of cues-to-action, such as a formal appointment reminder system. However, both healthcare providers and women indicated that healthcare providers sometime contact women with a missed appointment via phone or through social networks including family members. Healthcare providers pointed out that these approaches are time consuming and done irregularly. Women diagnosed with a high-risk condition and perceived this as severe, attended ANC due to their concerns. As one midwife pointed out, *“…they feel it is important for them, they write the date on their mobile so that they don’t forget it”.*

Regarding self-efficacy, almost all women said that they independently decided to attend ANC, and that they were confident about their choice. They also reported that they have adequate social capital to do so. One interviewee said, “*Inshalah, since I am educated, I can do the right thing*.”

### Pregnant women’s awareness, health information sources, and counseling

All pregnant women had heard of anemia, HDP, GDM and FGR. However, their awareness and descriptions of causes and consequences, varied based on the level of education, parity, personal history and knowing someone with at least one of the conditions. Women diagnosed with a condition were more aware of that condition and followed its progress more closely, compared to women not diagnosed with any of the high-risk conditions. Most of the diagnosed women remembered lab results, such as hemoglobin levels over time (Additional file 3 ^a^).

Healthcare providers, especially MCH doctors, were the main trusted sources of pregnancy related information. Several women also use the internet or ask their mothers if they need the information immediately. If they do not understand or find contradictory information, they prefer to confirm it with a doctor (Additional file 3 ^b^).

Most women felt that they received adequate health information during ANC visits (Additional file 3 ^c^). Women diagnosed with one of the high-risk conditions received information regarding that specific condition. Healthcare providers stated that they spent less time on counseling and health education than desired, due to the high patient load (Additional file 3 ^d^). Some pregnant women who were diagnosed with a high-risk condition (Additional file 3 ^e^) confirmed this.

### Attendance at ANC

Almost all women stated that they visited the health facility when they missed a period, and most healthcare providers had observed an increasing trend in early initiation of ANC. Most women were committed to scheduled ANC visits, but the degree of adherence differed based on their background characteristics, such as education, parity, and previous adverse pregnancy outcomes (Additional file 3 ^f^). Healthcare providers also said that most women are committed to scheduled ANC visits, including those with low-risk pregnancies. Women showed an interest in more frequent visits than currently recommended (Additional file 3 ^g,h^).

In sum, the in-depth interviews indicated that many women had limited knowledge about pregnancy related anemia, HDP, GDM, and FGR, and perceived the susceptibility to, and severity of, such conditions as low. While the general motivation to attend ANC was high, awareness of the importance of timely attendance to be appropriately screened and managed for such conditions was low. Women with high exposure to information about such pregnancy-related complications and the role of ANC (i.e. complications in current or prior pregnancy, knowing someone who experienced it, etc.) had high awareness of both the susceptibility to and severity of the conditions, as well as the benefits of timely ANC.

### Part II: Composing the text messages

We used the findings from the in-depth interviews above to identify gaps in information and awareness, across the HBM constructs, to be addressed in the TCC intervention. The national ANC guidelines recommend specific interventions towards the prioritized pregnancy related conditions in ANC visits at specific gestational ages (Additional file 1). We identified the five sentinel visits, corresponding to the specific time windows, as an opportunity to provide timely and actionable text messages based on the Model of Actionable Feedback. Actionable reminders of scheduled sentinel visits were combined with information about the susceptibility to and severity of the prioritized pregnancy related condition to be addressed, and the benefits of attending this sentinel visit. The text messages were co-designed iteratively with users. The Enhanced Active Choice, nudging theories and the Model of Actionable Feedback guided the framing, composition and timing of each text message.

### Intervention structure: target condition, frequency, timing and intensity

We developed text messages to be delivered in concert with the five sentinel recommended visits (gestational week < 16, 18–22, 24–28, 32 and 36) [34] in addition to a welcome message sent at the time of enrollment. The content of the text message corresponding to each sentinel visit was, among other factors, determined by the prioritized pregnancy related condition to be addressed through a screening test at that visit (Additional file 1). We applied the following message types and frequencies per visit:

1. One week before a scheduled sentinel visit: Women with a scheduled sentinel visit within an appropriate gestational age window, described above, receive this message. The message content addresses the benefit of attending ANC and the susceptibility to and, severity of the specific high-risk condition to be screened for, at that visit, according to national guidelines.
2. Three days before scheduled sentinel visit: Women with a scheduled sentinel visit and only with risk factors for the high-risk condition receives this message. The content addresses the increased susceptibility to, and the benefit of ANC attendance to be screened for timely. We use “scaled intensity” to intensify the intervention for those with the highest needs.
3. 24-h before any scheduled visit: All women with a scheduled visit irrespective of the gestational age receive this simple reminder.
4. 24-h after missed appointments for a sentinel visit: Only women with a missed appointment for a sentinel visit receive this reminder to re-schedule the appointment.

**Fig. 1:**
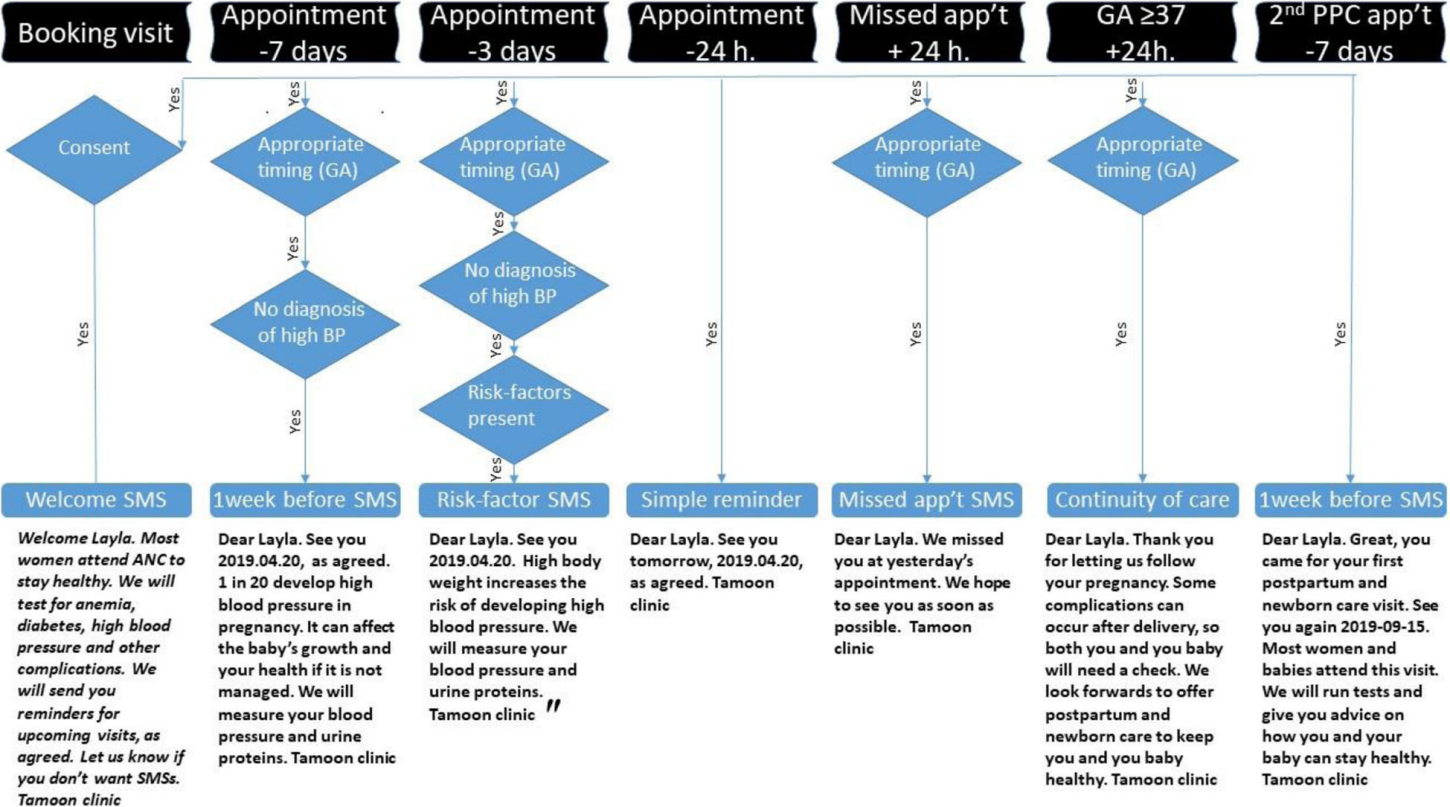
SMS library example for a low risk overweight woman targeting hypertensive disorders in pregnancy. 24-h before appointment is sent for all scheduled appointments, including PNC appointments. A week before and risk-factor SMS differ for each of the five sentinel visits, as indicated in Additional file 1

The package of text messages included continuity of care and postpartum care messages (Fig. 1). To illustrate, a woman above 34 years of age without a diagnosed high-risk condition, will receive 19 text messages if she starts ANC before gestational week 15, and is scheduled for and attends all five sentinel visits (Fig. 1). Missed appointment reminders would be additional.

### Content and framing of the text messages

The content in the text messages addressed key themes within the HBM constructs identified in Part I (Table 2). To address gaps in perceived susceptibility, the text messages included information about a woman’s relative chance of getting each high-risk condition (Table 2). We followed message-framing concepts and expressed proportions in an “x in y” format. For women with risk factors, we stated the risk factor and the increased susceptibility to the corresponding high-risk condition.

**Table 1:** Health Belief Model constructs including the main themes with quotes from the participants, Palestine

Model constructs	Description of the themes	Example excerpts
Perceived susceptibility	Knowledge: Susceptibility is perceived as higher among women who know the complications that can develop in pregnancy	“I don’t know about the disease, so how can I know if I am susceptible to it or not.” - *a primigravida*
	Self-care: Women perceive lower susceptibility as they engage in preventive self-care (e.g. healthy diets) and follow recommendations of care providers (e.g. ANC and screening)	“No, because I came to the clinic every time, and they [healthcare providers] reassured me that I didn’t have anything worrying. Also, in fact, I do not like sweets and sugar.” *- a primigravida* “I do not think so, because I am eating a good diet. As long as you have a good diet and milk and your hemoglobin is 12 and you are at the end of your pregnancy…” - *a low risk woman*
	Pregnancy history: Women with complications in previous pregnancies perceive themselves as more susceptible	“Yes, I had it [hypertension] in my first pregnancy and I recovered after delivery. Yes, I am susceptible because…” - *a woman who had a history of hypertension*
	Family history: Women with a family history of pregnancy complications or chronic conditions perceive themselves as more susceptible	“No I don’t worry, and there is nobody in my family who has diabetes” - *a hypertensive woman* “I don’t know exactly, my parents don’t have hypertension and my husband’s parents have hypertension, so may be my children will have hypertension in the future.” - *a pregnant woman who had miscarried five times*
Perceived severity	Chronic conditions, not pregnancy complications. Women relate their perception of severity to the conditions as chronic conditions, but not their potential for complicating pregnancy	“I know that diabetes delays healing of the wound and this may cause amputation of limbs…” *- a woman attending a high-risk clinic* “Heart problems and increase heart rate, dizziness and loss of consciousness” - *a low-risk woman* “I do not know if it affects [the baby]” - *a primigravida with moderate anemia*
	History of friends/relatives: Women who know friends/family with a history of pregnancy complications perceive complications as more severe	“…hypertension is dangerous for pregnant women and leads to preeclampsia, I know a friend who had eclampsia at the end of the eighth month” *- a multigravida* “My sister had anemia and her hemoglobin became 5, and she needed two units of blood…” - *a grand multipara*
	Being affected by a complication: Women diagnosed with a high risk condition, often articulate clearly the potentially severe consequences of the condition	“Premature baby, low birth weight or IUGR” - *a pregnant woman in a high-risk clinic* “It can cause early labor, bleeding and thrombosis” - *a woman with coagulation disorder*
Perceived benefit	Expectations to care content: Advance knowledge of purpose and what tests each scheduled visit would include, affects the women’s perception of benefit	“I found that [private] doctor and [public] clinic providing the same services, such as weight, height, blood pressure measurements, so I decided to follow up in the [public] clinic” - *a pregnant woman at low-risk clinic trying out services in Gaza* “I have to come. It is my duty to come for ANC visit” *- a primigravida*
	Being affected by a complication: Women diagnosed with a high risk condition perceive the importance of visiting the clinics according to the schedule, but only for the specific condition they are diagnosed with	“…examine the level of sugar and control…” *- a woman attending a high-risk clinic* “I follow my periodic check-ups every month …I receive the anticoagulant injections…” *- a woman with coagulation disorder* “Of course it is beneficial, since I get the anti-hypertensive drugs, iron and vitamins” - *a woman diagnosed with HDP*
Perceived barrier	Perception of benefits: The better the perceived benefit the woman have, the less perceived barrier to attend the scheduled visits	“I think that there are no obstacles, and I should follow the right things for my benefit.” *- a primigravida* “I think, there are no difficulties, and the most important thing is having personal will” *- a grand multipara*
	Family logistics: Women with small children and little family support, report this as a barrier to attend ANC	“In the first and second pregnancies, I attended regularly, but when the number of my children increased, it became less often than before.” *- a mother of three* “…my children are small and my husband works in military and he comes back at night…” *- a multipara*

We stated the consequences of each high-risk condition to both the woman and her baby to address the perceived severity, given that most women knew the general population consequences of the chronic disease, but not the specific adverse effects during gestation. We avoided serious and grave consequences, such as death or malformation, to prevent unwarranted worries in pregnancy. We presented the potential screening tests that could detect each high-risk condition to address the perceived benefits (Additional file 1).

**Fig. 2:**
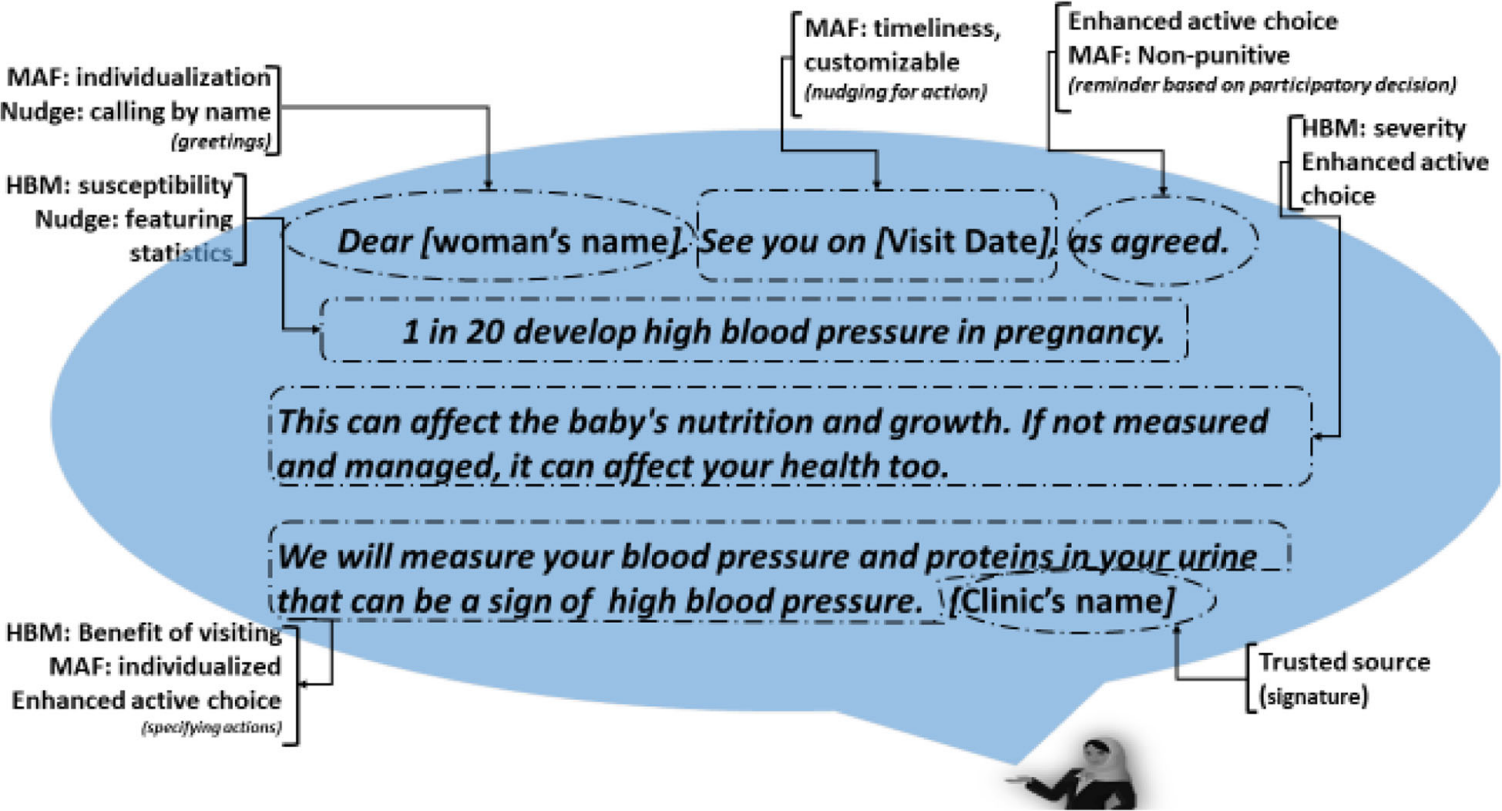
An example of how the Health Belief Model (HBM), Model of Actionable Feedback (MAF), Enhanced Active Choice (EAC) and social nudging theories were used to compose the text messages. [Woman’s name] the algorithm pulls the first name of the recipient and inserts here. [Visit Date] the date of scheduled visit appears in YYYY.MM.DD format automatically. [Clinic’s name] the name of public primary health care where the woman attends her visits automatically inserted

The Model of Actionable Feedback [36] and concepts of social nudging and Enhanced Active Choice [11] were used to structure the text messages, and make them action oriented while addressing knowledge gaps, beliefs and perceptions (Fig. 2, Additional file 2). Concepts from the Model of Actionable Feedback, including timeliness, non-punitiveness, customizability, and individualization, were among the guiding principles at each stage of the TCC content development, evaluation, and implementation. See Additional file 2 for details on the application of the Model of Actionable Feedback and Enhanced Active Choice in composing the text messages.

### Evaluation of the draft text messages

Generally, the content of the text messages was understandable and acceptable after evaluation by health educators, healthcare providers, and pregnant women. Minor changes to a few messages were made based on comments from stakeholders. Contextualized translation into Arabic was preferred over literal translation. Regarding the timing, women preferred to receive the text messages after working hours when they are free to read the text messages.

## Discussion

In co-design with users, we developed a TCC intervention tailored to individuals to be sent via SMS. We underpinned our intervention with behavior change theories and recommendations from systematic reviews [13, 14, 17, 18], and composed messages following recommended framing models [11, 37]. The message development process was iterative, and we involved end-users from the beginning. The MCH eRegistry [26, 27] presents a unique opportunity for an individually tailored and automated TCC intervention development.

Our qualitative findings revealed that perceived susceptibility, severity, and benefits are the main HBM constructs affecting whether or not pregnant women attend ANC for screening and management of high-risk conditions (anemia, HDP, GDM, and FGR) in a timely fashion. This is in line with systematic reviews of ANC service utilization, reporting that women’s perceptions of risks (perceived susceptibility and severity) and the ANC service’s potential to identify and manage risks (benefits), affect timely attendance [38, 39]. Access to health care facilities was not reported as an important barrier to attend ANC in our sample. However, it has been reported that the presence of checkpoints restricted access to health facilities in Palestine [40], and one of the major factors in other settings [38, 39].

Analyses guided by the HBM assisted us in identifying information targets to be addressed in the TCC intervention. Our TCC intervention directly targets perceived susceptibility, severity, and benefits, and indirectly targets perceived barriers, cues-to-action and self-efficacy. Through SMS, we aim to improve the awareness of personal susceptibility to, and severity of high-risk conditions, and the benefit of timely attendance to ANC for screening and management. We believe that these text messages from the eRegistry can serve as cues-to-action and affect self-efficacy by empowering women.

Among behavior change theories used for similar interventions [22], we selected the HBM as theoretical framework based on its common use in similar resource-limited settings [24]; the nature of our behavior change goal; the scope of our intervention; and our target audience [22]. The HBM served as a lens to understand the context and to identify the major gaps to be addressed. We also used social nudging, a concept derived from behavioral science, and the Enhanced Active Choice model for framing the text messages [11, 37]. We wanted women to make a conscious decision regarding ANC attendance. Information about the gains and losses of timely attendance was used to nudge women to attend timely scheduled visits [11]. The Model of Actionable Feedback was used to help transition women from intention to actual performance of the expected behavior [36]. It guided, for example, the insertion of the scheduled date right after the personalized greetings, since the action or targeted behavior change goal, is to attend the facility in a timely manner. All the different components in a text messages were created based on the concepts and theory we used (Fig. 2).

Limited information exists regarding the best processes for designing the content of a TCC intervention. It is recommended to publish the development process, as well as the effectiveness of digital health interventions [41], to increase scientific discussions and improve the quality of digital health interventions including TCC. We have considered learning points from previous development processes despite the differences in technologies used [23, 41, 42]. Communicating back to the client from the registries is one of the functions which can be utilized better [26]. We demonstrate how longitudinally collected digital data can be harvested to deliver more personalized health education and promotion messages in the growing field of mobile technology for health, in an integrated way with the health systems infrastructure.

### Strengths and limitations

Our text messages are based on modifiable factors among the recipients by our digital intervention, such as gaps in knowledge and awareness. We have refrained from sending condition-specific messages to woman diagnosed with the same high-risk condition, i.e. a woman with hypertensive disease in pregnancy will not receive messages about her susceptibility to develop hypertension in pregnancy. This includes avoiding sending messages that included recommendations for action to women with high-risk conditions managed in high-risk clinics, where management may be highly individualized and not determined solely by guideline-driven algorithms.

We addressed confidentiality issues concerning the TCC content throughout the design process. Women might share their phones with other family members, and someone other than herself might therefore see the text messages. We have therefore de-sensitized the TCC content by not indicating that the receiver has a specific risk factor. For example, for a woman with high BMI, we phrased the message as “…*High weight increases the risk of diabetes. We will measure your…”* without directly stating that she is obese*.*

The main project, aimed at assessing the effectiveness of automated TCC in improving ANC and PNC attendance, pioneers the use of the DHIS2 Tracker software to send tailored text messages on specific dates and times at national scale. This involves scheduling of messages based on data, a functionality that did not exist in the software at the outset of the project, and therefore required longer development and enhancement time than first anticipated. Future projects should take the readiness of the technology and the time needed for software development into account when planning the overall timeline. It is a challenge to express necessary and personalized information in a clear and understandable way within the limitations of SMS messages (size of screen, acceptability, and cost increasing by “one SMS” for each 160 characters in Latin letters [43] and 70 characters in Arabic). The fact that several of our messages have over 140 Arabic characters (i.e. cost as for three messages), may affect the sustainability if used in a national system. We have not utilized the full potential of the current technology due to development needs, but further tailoring to various static and dynamic personal characteristics should be possible with the MCH eRegistry. Additionally, two-way communication has been shown to be more effective than a one-way messaging [44]; this feature is not yet routinely available in the software.

There is no one-size-fits-all approach for deciding the ideal ‘dosage’ of text messages to be delivered to an individual to change behavior. The threshold is likely to vary according to the richness of the content, intervention type, and target group [45, 46]. We have chosen our dosage after discussing with the local experts, but have not put the final quantity under formal evaluation. We selected the timing by considering the balance between the time needed to prepare for a visit (one week ahead as a cue to action and enable reflection and planning of practicalities) and not forgetting the date of that visit (24 h before as a simple reminder). However, most untailored TCC interventions in the field of maternal and child health, has reported sending more text messages per week than we propose [13, 15, 47].

## Conclusions

The stepwise iterative process revealed elements critical to an effective TCC intervention, which otherwise could have been easily missed. The theory served as a lens through which we assessed gaps in anticipated behavior, a focus in composing the text messages, and a guide in overall designing and implementation of the TCC intervention. Behavioral science concepts made us value each word and its relative position in the text. The co-design with users improved the relevance, understandability, and acceptability of the text message content. The MCH eRegistry can serve as a unique platform through which to tailor communication. Reporting on the development process of our TCC intervention will improve transparency and contribute to scientific dialogue to improve its effectiveness.

## Supplementary information

**Additional file 1.** The timing and services provided according to the Palestinian national antenatal guidelines and the corresponding high-risk conditions addressed in the tailored Targeted Client Communication (TCC) intervention

**Additional file 2.** Theories and concepts used in the design of the Targeted Client Communication (TCC) intervention

**Additional file 3.** Quotations from the pregnant women and healthcare providers from the in-depth interviews, Palestine

## Abbreviations

ANC: Antenatal Care
FGR: Fetal Growth Restriction
GDM: Gestational Diabetes Mellitus
HBM: Health Belief Model
HDP: Hypertensive Disorders of Pregnancy
MCH: Maternal and Child Health
mHealth: Mobile Health
PNC: Postnatal Care
SMS: Short Message Service
TCC: Targeted Client Communication
WHO: World Health Organization
