# Supplementary material for: Development of a targeted client communication intervention to women using an electronic maternal and child health registry: a qualitative study
Source: BMC Med Inform Decis Mak. 2020 Jan 6;20:1. doi: 10.1186/s12911-019-1002-x (PMC6945530; doi:10.1186/s12911-019-1002-x)
Supplement: Supplementary file 1 — Additional file 1. The timing and services provided according to the Palestinian national antenatal guidelines and the corresponding high-risk conditions addressed in the tailored Targeted Client Communication (TCC) intervention [file 12911_2019_1002_MOESM1_ESM.docx]

# **Additional file 1: The Palestinian national antenatal guidelines and the tailored Targeted Client Communication (TCC) intervention**

| Five sentinel Antenatal Care visits | High-risk conditions and recommended screening tests | Timing of the text message ^+^ | TCC content including prioritized high risk conditions |
| --- | --- | --- | --- |
| Booking visit  (as early as possible) | Anemia: Hb  HDP: BP, urine test for protein  GDM/DM: urine test for glucose  FGR: ultrasound, or fundal height for late booking visits | NA | Welcome and introduction to the aim of the TCC intervention |
| 1^st^ visit  Gestational Week 16 (GW 16) | FGR: fetal growth monitoring (fundal height measurement, from GW 16)  Routine vital sign follow-ups  Other examinations up on indications | [15-17] GW | ***Fetal growth and wellbeing***: stating the importance of pregnancy follow-ups, specifically focusing on the importance of early diagnosis for anemia ( as a risk factor for FGR) and other risk-factors for FGR for, when relevant |
| 2^nd^ visit  (18-22 GW) | HDP: routine BP measurement  Urine test for protein  FGR: regular ultrasound  Other examinations up on indications | [18-22] GW | ***Hypertension:*** creating awareness about the importance of regular screening for elevated blood pressure and urine protein to diagnose for preeclampsia/ pregnancy induced hypertension and risk-factors for HDP |
| 3^rd^ visit  (24-28 GW) | GDM: Random/fasting blood glucose  Anemia: Hb  Routine vital sign follow-ups  Other examinations up on indications | [24-28] GW | ***Diabetes*** and ***Anemia:*** focused persuasive messages and reminders for the lab investigations and risk-factors for GDM |
| 4^th^ visit  (GW 32) | FGR: fetal growth monitoring (fundal height measurement)  Routine vital sign follow-ups  Other examinations up on indications | [31-33] GW | ***Fetal growth and wellbeing***: stating the importance of continues ANC visits to follow the babies growth, and early detection of hypertension as a risk factor for FGR |

ANC = Antenatal Care; BP = Blood Pressure; GW = Gestational Week; Hb = Hemoglobin; HDP = Hypertensive Disorder of Pregnancy; GDM = Gestational Diabetes Mellitus; DM = Diabetes Mellitus; FGR = Fetal Growth Restriction; NA = Not Applicable
