# Supplementary material for: Development of a targeted client communication intervention to women using an electronic maternal and child health registry: a qualitative study
Source: BMC Med Inform Decis Mak. 2020 Jan 6;20:1. doi: 10.1186/s12911-019-1002-x (PMC6945530; doi:10.1186/s12911-019-1002-x)
Supplement: Supplementary file 2 — Additional file 2. Theories and concepts used in the design of the Targeted Client Communication (TCC) intervention [file 12911_2019_1002_MOESM2_ESM.docx]

# **Additional file 2: Theories and concepts used in the design of the Targeted Client Communication (TCC) intervention**

| Theories/  concepts | Components | Theoretical assumptions | Theories/concepts guided the development of the TCC content to be sent via SMS |
| --- | --- | --- | --- |
| Model of Actionable Feedback (MAF) | Timeliness | The recipient has to receive the SMS at an appropriate time to be able to act upon it | - All the SMSes were sent within one week of the scheduled visits (intended behavioral action),to empower women to attend the Antenatal Care visit in a timely manner - States the exact date of the scheduled/missed antenatal care visit in the SMS - Use the most recent information, i.e. in the MCH eRegistry - The eRegistry was scheduled to send the SMS at 17:00 hour, informants recommended this time |
|  | Individualization | The recipient will understand the content of the SMS and be able to act upon it, if it is specific for her | - Tailored to individuals based on gestational age, selected risk-conditions(anemia, hypertension, diabetes, and fetal growth restriction), and selected risk factors (age, BMI, previous hypertension, diabetes, and anemia) to the risk-conditions risk factor messages will only be sent the woman with specific risk factor/s - States the recipient’s name in a direct speech - Directed to the recipient as an individual, not generic contents |
|  | Non-punitive | The recipient will change behavior if the instructions are communicated with positive reinforcement | - Positive tone; not dictating from top down; states mutual agreement - States that the woman participated in the decision (e.g. phrase like “*as agreed*” was included in the messages), empowering - Does not imply negative consequences in future ANC services if they fail to attend. - Serious losses, e.g. death of the baby, were not mentioned |
|  | Customizable | The recipient will find the information in the SMS meaningful and acceptable if it is customized for her | - Each recipient receives the messages pertaining her conditions - Any dynamic variable (gestational age, risk-status, risk-factors) are customized based on the routinely collected data at each visit - Name of the recipients and name of the clinic as a sender |
| Behavioral science concepts | Enhanced Active Choice | The recipient will be empowered to make consciously informed decisions/ informed health choices and commit to them if they are provided with benefits of adopting the instructions, and consequences of not. | - Provides information about the benefits of timely attendance and consequences of not attending timely. Does not state fear-inducing or loss-framed content to avoid side-effects (pregnancy worries) |
|  | Calling by name | The recipient’s name is preferable in communication | - First name of a recipient is included - Culturally acceptable greeting |
|  | Using “trusted sources” as a sender | The recipient will welcome and accept the SMS if it is signed off by a trusted sources | - The name of the recipient’s primary healthcare clinic is included as a sender |

BMI: Body Mass Index; SMS: Short Text Message; TCC: Targeted Client Communication
