# Supplementary material for: Development of a targeted client communication intervention to women using an electronic maternal and child health registry: a qualitative study
Source: BMC Med Inform Decis Mak. 2020 Jan 6;20:1. doi: 10.1186/s12911-019-1002-x (PMC6945530; doi:10.1186/s12911-019-1002-x)
Supplement: Supplementary file 3 — Additional file 3. Quotations from the pregnant women and healthcare providers from the in-depth interviews, Palestine [file 12911_2019_1002_MOESM3_ESM.docx]

# **Additional file 3: Quotations from the pregnant women and healthcare providers, from an in-depth interview, Palestine.**

| Reference in the text | Quotations |
| --- | --- |
| ^a^ | “My hemoglobin is now 9.1 g/dl, and my ferritin level is low. I am currently taking iron tablets, but, I can’t feel that I am benefiting from them”   - *A multigravida from the West Bank* |
| ^b^ | “I will follow my doctor’s opinion because she has more experience than others” A primigravida woman |
| ^c^ | “Of course, they [healthcare providers] provide all the information and advices I need during pregnancy, including how to deal with any problem when it happens. In addition, they gave me the appropriate medications.”   - *A multigravida in a low-risk clinic* |
| ^d^ | “We counsel them, but when I have 20 pregnant women in a day … I try to explain quickly, but I feel that there is not enough time per client for counseling”   - *A doctor* |
| ^e^ | “There is no enough time because of the overload of pregnant women in the clinic, in general, but when I need important information, I ask the doctor in the clinic and I get what I need”   - *A multigravida in a high-risk clinic* |
| ^f^ | “…Previously, they used to come when they were two or three months pregnant. As a doctor, [I know that] they miss a very important period in their pregnancy. Now women come early and they are aware of the importance of early booking”   - *MCH doctor* |
| ^g^ | “Yes, they feel that it is important for them, they write the date on their mobile so that they cannot forget it, and they [pregnant women] complain of the new guidelines since there is a distance between each visit for normal pregnancies.”   - *A midwife* |
| ^h^ | “Yes, thank God, but the timing between the visits are long. It is long to wait for one month to see your health professional”   - *A multigravida woman* |
